# External Validation of the RETREAT Score for Prediction of Hepatocellular Carcinoma Recurrence after Liver Transplantation

**DOI:** 10.3390/cancers14030630

**Published:** 2022-01-27

**Authors:** Maria C. van Hooff, Milan J. Sonneveld, Jan N. Ijzermans, Michail Doukas, Dave Sprengers, Herold J. Metselaar, Caroline M. den Hoed, Robert A. de Man

**Affiliations:** 1 Department of Gastroenterology and Hepatology, Erasmus MC Transplant Institute, University Medical Center Rotterdam, P.O. Box 2040, 3000 CA Rotterdam, The Netherlands; m.vanhooff@erasmusmc.nl (M.C.v.H.); m.j.sonneveld@erasmusmc.nl (M.J.S.); d.sprengers@erasmusmc.nl (D.S.); h.j.metselaar@erasmusmc.nl (H.J.M.); c.denhoed@erasmusmc.nl (C.M.d.H.); 2 Department of Surgery, Division of HPB & Transplant Surgery, Erasmus MC Transplant Institute, University Medical Center Rotterdam, P.O. Box 2040, 3000 CA Rotterdam, The Netherlands; j.ijzermans@erasmusmc.nl; 3 Department of Pathology, Erasmus MC, University Medical Center Rotterdam, P.O. Box 2040, 3000 CA Rotterdam, The Netherlands; m.doukas@erasmusmc.nl

**Keywords:** hepatocellular carcinoma, liver neoplasms, liver transplantation, Milan criteria, risk score, recurrence

## Abstract

**Simple Summary:**

Liver transplantation (LT) is a potentially curative treatment option for hepatocellular carcinoma (HCC), but is frequently complicated by HCC recurrence. In order to estimate the recurrence risk a novel risk score was developed in the United States: the Risk Estimation of Tumor Recurrence After Transplant (RETREAT). External validation of this novel risk score, in a different patient population with other LT selection criteria, is needed. In this study we demonstrate that the RETREAT score is able to predict the risk of HCC recurrence after liver transplantation in a European population. These findings may be used to inform patients of recurrence risk and as a basis for studies on surveillance strategies.

**Abstract:**

Background. We aimed to externally validate the performance of the RETREAT score in a European population. Methods. This single center retrospective cohort study enrolled all consecutive patients with HCC who underwent LT between 1989 and 2019. The performance of RETREAT was assessed in the overall population and after stratification between being within or beyond the Milan criteria based on the explant pathology report. Recurrence probabilities were estimated by using the Kaplan–Meier method and compared by log-rank test. Results. We studied 203 patients; 42 patients were beyond the Milan criteria based on explant pathology. The median follow-up was 26.8 months (IQR 7.2–60.7). Overall cumulative HCC recurrence rates were 10.6%, 21.3%, and 23.0% at 2, 5, and 10 years, with the majority of recurrences extrahepatic and at multiple sites. Higher RETREAT scores were associated with higher recurrence rates, with a 10-year recurrence rate of 60.5% in patients with RETREAT ≥ 3 (*n* = 65), compared to 6.2% in those with RETREAT ≤2 (*n* = 138; *p* < 0.001). HCC recurrence rates were even lower in patients within the Milan criteria who also had a low RETREAT score (*n* = 122; 2.7% at 10 years). Conclusion. Low RETREAT scores identify patients at low risk of HCC recurrence after LT in patients within the Milan criteria based on explant pathology.

## 1. Introduction

Liver transplantation (LT) is an important potentially curative treatment option for patients with early stage hepatocellular carcinoma (HCC) [1]. Selection for LT in the Eurotransplant region is based on the Milan criteria (MC), limiting eligibility to patients with a single tumor less than 5 cm in size, or up to three tumors all smaller than 3 cm [2,3]. However, recurrence of HCC after LT occurs in approximately 8–20% of patients despite application of these stringent selection criteria [2,4,5,6,7]. Previous studies suggested that up to 75% of the tumor recurrence occurs during the first two years after LT with a median survival after recurrence of 7 to 16 months [4,5].

Over the past decade, several groups have described risk factors for HCC recurrence after LT. A recent review identified several predictors of HCC recurrence, which include: patient related factors (underlying disease and hepatitis C virus treatment), the tumor related factors (tumor staging, vascular invasion, and differentiation grade), biomarkers (alpha-fetoprotein and the neutrophil lymphocyte ratio), radiological factors and treatment related factors (time on the waiting list, pre-transplant bridging therapy, and response to locoregional treatment (LRT)) [4,8]. Various groups have applied these factors to construct prediction models which could potentially be used to stratify recurrence risk. 

The best characterized postoperative prediction model is the Risk Estimation of Tumor Recurrence After Transplant (RETREAT) score [9,10], which is based on the sum of the largest viable tumor diameter and number of viable tumors on explant, the presence of microvascular invasion, and the alpha-fetoprotein (AFP) level at the time of LT. The performance of the RETREAT model has so far been assessed within the United Network for Organ Sharing (UNOS) Database (United States) and in three single-center studies [11,12,13,14].

The aims of this current study were to (1) quantify the risk of HCC recurrence in patients who underwent a LT for HCC in the Netherlands and (2) to assess whether the RETREAT score can be used to stratify recurrence risk in a European patient population.

## 2. Patients and Methods 

### 2.1. Patients

This single center retrospective cohort study included all consecutive patients with a LT for (suspected) HCC between October 1989 and October 2019 in the Erasmus MC University Medical Center in Rotterdam, the Netherlands. Patients were identified through a database search and variables were retrospectively collected from patient records.

Patients were excluded for this study if (1) there was no evidence of HCC on explant in patients who had not received locoregional therapy (LRT), (2) if explant pathology showed cholangiocarcinoma, mixed hepatocellular cholangiocarcinoma, or only benign lesions, (3) if there were insufficient data to calculate RETREAT score, (4) if no follow-up data post-LT were available, and (5) if patients developed a recurrence in the pre-LT liver biopsy tract. Follow-up was completed until December 2019. 

All patients provided written informed consent before LT approving the use of their data for research purposes. The current study was approved by the medical ethical committee of the Erasmus MC University Medical Center (Study identifier MEC-2019-0775) and was conducted in accordance with the principles of the Declaration of Helsinki. 

### 2.2. Post-Transplant Follow-Up and Diagnosis of HCC Recurrence

Follow-up after LT consists of regular visits to the outpatient clinic, monitoring of AFP, biannual ultrasound, and annual chest x-ray. Additional liver or whole body imaging was performed according to patient and physician preferences and guided by symptoms and/or elevated AFP. Diagnosis of HCC recurrence was based on histopathology reports or clinical consensus.

### 2.3. Milan Criteria on Explant and RETREAT Score

All patients were classified as within or beyond the MC based on the explant pathology report.

The RETREAT score was calculated for each patient as previously reported based on pre-transplant AFP and explant histopathological features: presence of microvascular invasion and largest viable tumor diameter (cm) plus number of viable tumors. A patient’s RETREAT score can range from 0 to a maximum of 8 points [10]. Patients with an AFP < 20 ng/mL, no microvascular invasion on pathology, and completely necrotic tumor(s) on explant after LRT, are given 0 points. 

### 2.4. Statistical Analysis

SPSS version 26 was used for statistical analyses. Data are presented as either mean (SD) or median (interquartile range, IQR) where appropriate. Associations between variables were tested using Student’s *t*-test, Chi-square, Pearson correlation, or their non-parametric equivalents, when appropriate. 

Recurrence probabilities were estimated (at 2, 5, and 10 years) using the Kaplan–Meier method. Recurrence probabilities were compared for patients within and beyond the MC (based on explant pathology) and across RETREAT scores with log-rank tests. In addition, performance of the RETREAT was also assessed after stratification by being within or beyond the MC on explant pathology. Patients lost to follow-up were censored at the time of the last tumor free visit. Diagnostic performance was assessed using sensitivity, specificity, and negative predictive values (NPV) using the entire follow-up period. In addition overall survival probabilities were estimated using the Kaplan–Meier method and compared by log-rank test for low and high risk groups.

## 3. Results

### 3.1. Patient Characteristics

A total of 203 patients were eligible for analysis (Supplementary Figure S1). The characteristics of the included patients are shown in Table 1. The median time on the waiting list was 7.7 months (IQR 3.8–10.8). All patients were within the Milan criteria on last imaging pre LT. The median follow up time after LT was 26.8 months (IQR 7.2–60.7). Prior to LT, 153 of the 203 patients (75.4%) underwent LRT. Locoregional therapy mainly consisted of a liver resection (*n* = 17 (8.4%)), microwave ablation (MWA) (*n* = 20 (9.9%)), radiofrequency ablation (RFA) (*n* = 95 (46.8%)), or transarterial chemoembolization (TACE) (*n* = 71 (35.0%)) (Table 1). In over 45%, the underlying liver disease was viral hepatitis alone or in combination with other chronic liver diseases. A total of 42 of the 203 patients (20.7%) were beyond the MC based on explant pathology.

### 3.2. HCC Recurrence

**Figure 1:**
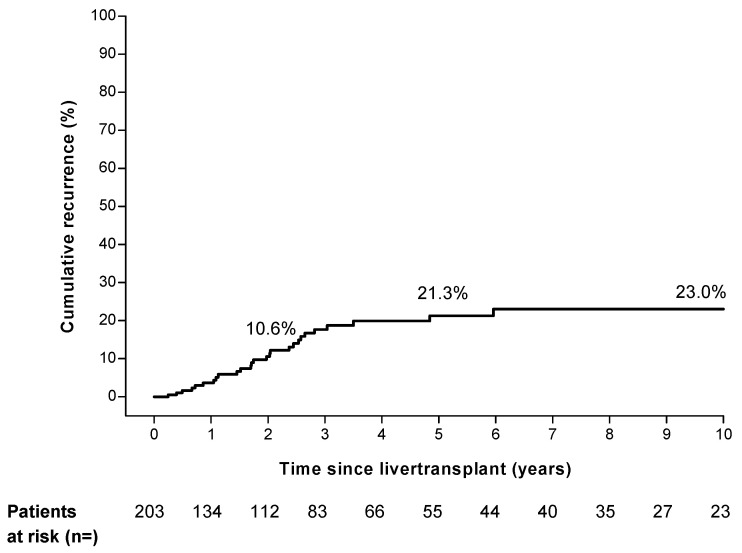
Overall hepatocellular carcinoma recurrence risk in study cohort.

In our cohort, 27 of the 203 patients had a recurrence of HCC after LT. The median time to recurrence was 20.5 months (range 2.9 to 71.5 months). The estimated cumulative rate of HCC recurrence was 10.6% at 2 years, 21.3% at 5 years, and 23.0% at 10 years (Figure 1). Twelve of the twenty-seven (44%) recurrences of HCC occurred beyond 2 years after LT. Most of the recurrences were located in the liver (51.9%), lung (48.1%), and bone (22.2%) (Supplementary Table S1). In 14 of the 27 cases (52%) recurrence occurred at multiple sites simultaneously. Fifteen of the twenty-seven patients received oncologic treatment after recurrence (Supplementary Table S1). The 3, 6, and 12 month cumulative mortality after recurrence was 18.5%, 29.6%, and 67.4%. 

### 3.3. Higher Recurrence Rate in Patients beyond Milan Criteria on Explant

**Figure 2:**
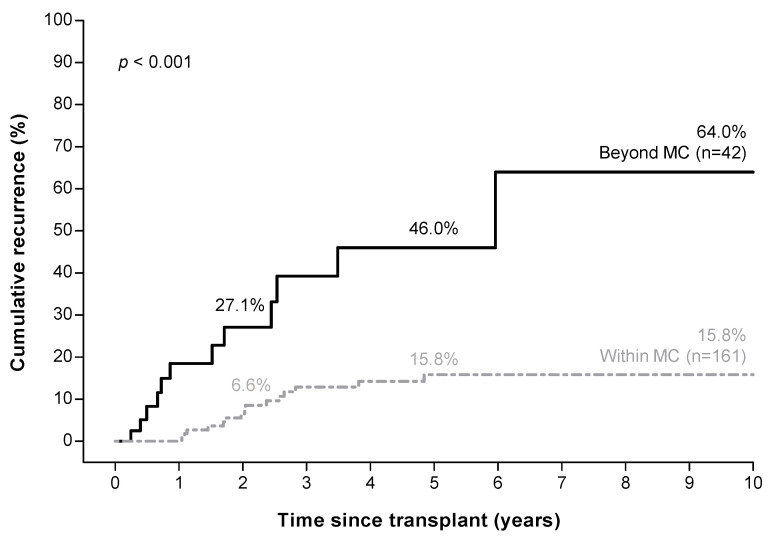
Higher hepatocellular carcinoma recurrence risk in patients beyond the Milan criteria ^a^ based on explant pathology. ^a^ Milan criteria (MC): one tumor ≤ 5 cm, or three tumors ≤ 3 cm each.

Forty-two (20.7%) patients were beyond the MC based on explant pathology. Patients beyond the MC had a recurrence rate of 27.1%, 46.0%, and 64.0% at 2, 5, and 10 years respectively, compared to 6.6%, 15.8%, and 15.8% in patients within the MC (*p* < 0.001, Figure 2).

### 3.4. RETREAT Score Predicts HCC Recurrence

The distribution of tumor characteristics for individual patients at the time of LT is shown in Table 2. The vast majority of the patients (78.8%) had an AFP below 20 ng/mL. A retreat score of 0 was found in 18.2% of the patients; 34.5% of the patients had a score of 1; 15.3 % a score of 2 points; 14.3% of 3 points; 9.4% of 4 points; and 8.4 % of 5 or more points. 

**Figure 3:**
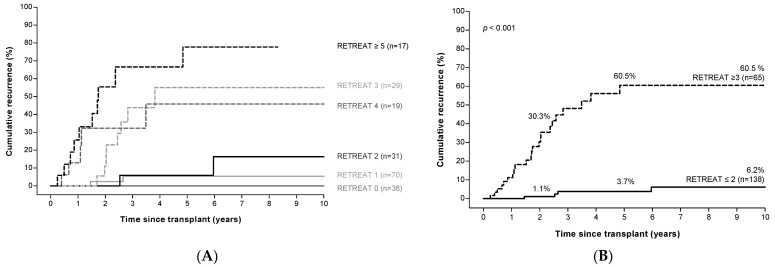
RETREAT ^a^ score predicts HCC recurrence risk after LT. Panel (**A**): Risk of HCC recurrence according to RETREAT score. Panel (**B**): Risk of HCC recurrence in patients with low versus high RETREAT scores. ^a^ RETREAT score: α-fetoprotein at LT, microvascular invasion, and sum of largest viable tumor diameter and number of tumors. Abbreviations: AFP, α-fetoprotein; HCC, hepatocellular carcinoma; LT, liver transplantation.

Higher RETREAT scores were associated with significantly higher HCC recurrence rates; 5-year cumulative recurrence rates were 0% in patients with a RETREAT score of 0, compared to 77.7% in patients with a RETREAT score ≥ 5 (Figure 3A). Combining patients with RETREAT scores of ≤2 identified a low-risk group (*n* = 138) with a cumulative recurrence risk of 1.1%, 3.7%, and 6.2 % at, respectively, 2, 5, and 10 years of follow up. 

Patients with a RETREAT score ≥ 3 (*n* = 65) were identified as a high-risk group with a cumulative recurrence rate of 30.3%, 60.5%, and 60.5% at 2, 5, and 10 years of follow up (Figure 3B).

### 3.5. RETREAT Score Stratifies HCC Recurrence Risk in Patients within the Milan Criteria

**Figure 4:**
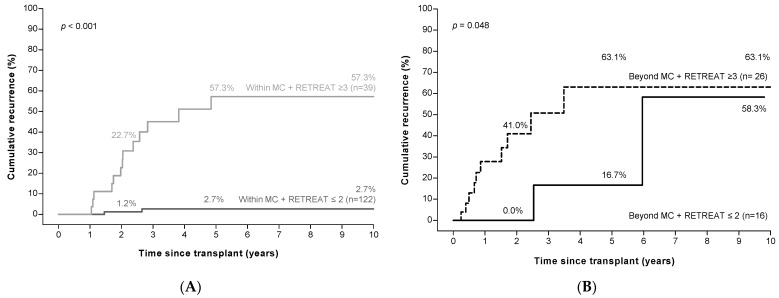
Low RETREAT scores ^a^ identify patients at low risk of HCC recurrence in patients within the Milan criteria ^b^ (panel (**A**)), but not in those beyond the Milan criteria (panel (**B**)). ^a^ RETREAT score: α-fetoprotein at LT, microvascular invasion, and sum of largest viable tumor diameter and number of tumors. ^b^ Milan criteria (MC): one tumor ≤5 cm, or three tumors ≤3 cm each. Abbreviations: HCC, hepatocellular carcinoma.

Among patients with HCC within the MC based on explant pathology (*n* = 161), 122 patients had a RETREAT score of ≤2. HCC recurrence was extremely rare in these patients (recurrence at 2, 5, and 10 years; 1.2%, 2.7%, and 2.7%), yielding a sensitivity of 92.6%, specificity of 68.2%, and a negative predictive value of 98.3%. Among the 39 patients with a RETREAT score ≥3, recurrence rates were high (recurrence at 2, 5, and 10 years; 22.7%, 57.3%, and 57.3%, Figure 4A). Recurrence risk in this group (within the MC, but with a high RETREAT score) did not differ from the 42 patients who were outside of the MC (*p* = 0.75, Supplementary Figure S2). Finally, among the 42 patients beyond the MC on explant pathology, the recurrence rate was high even in those with low RETREAT scores (Figure 4B). 

### 3.6. Overall Survival after Liver Transplantation Stratified by RETREAT

**Figure 5:**
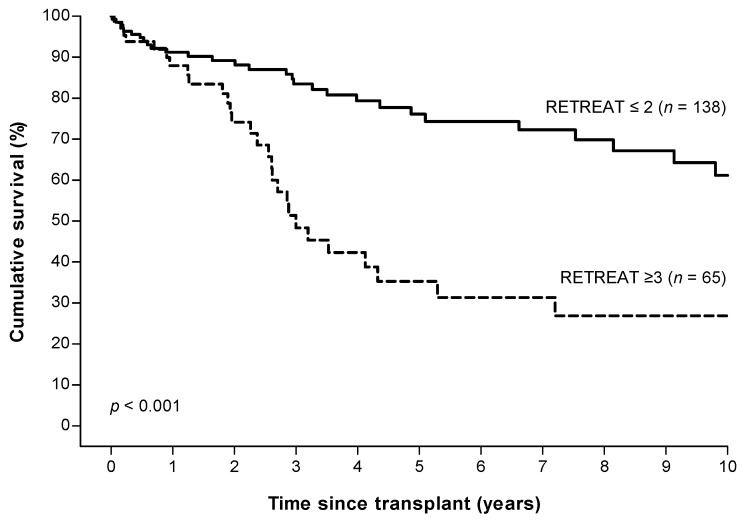
Overall survival in patients with low (≤2) versus high (≥3) RETREAT scores ^a^. ^a^ RETREAT score: α-fetoprotein at LT, microvascular invasion, and sum of largest viable tumor diameter and number of tumors.

The 2, 5, and 10 years overall survival after LT was 84.5%, 63.9%, and 50.8%. The 5 years overall survival for patients in the high risk group (RETREAT ≥ 3) was significantly lower compared to the low risk group (RETREAT ≤ 2) (35.3% vs. 76.1%, *p* < 0.001, Figure 5). 

## 4. Discussion

Most transplant programs apply stringent criteria to select patients with HCC for LT, in order to limit the risk of subsequent HCC recurrence. Nevertheless, HCC recurrence is observed in a considerable number of patients. In the current study we observed HCC recurrence in 23% of patients, with almost half of the cases diagnosed beyond two years after LT, and one recurrence even beyond five years post-LT. This finding contrasts previous reports, which suggested that approximately 75% of the tumor recurrence occurs during the first two years after LT, with a median time to recurrence of 12 to 14 months, compared to 20 months in our cohort [5,6,15,16]. These findings may be partly accounted for by tumor and treatment characteristics that may differ between cohorts, but they underscore the need for constant vigilance with regard to the risk of HCC recurrence.

Given the high risk of HCC recurrence, many centers apply surveillance programs, although their effect on outcomes is still uncertain. At present, there are no uniform recommendations on the mode and frequency and duration of surveillance [2,17,18]. This high variability in surveillance strategy was confirmed in a recent national survey study in the United States [19]. In our cohort, patients underwent surveillance consisting of an annual chest x-ray, biannual liver imaging by ultrasound, and regular blood tests. Additional imaging was performed at the discretion of the treating physician. In case of clinical suspicion of recurrence, extensive additional imaging with CT was performed. As shown in Supplementary Table S1, the majority of patients with HCC recurrence had extrahepatic recurrence, often at multiple sites. These findings suggest that if surveillance is performed, chest and abdominal imaging using CT or MRI may have the highest yield. Furthermore, it should be appreciated that curative therapy is often not possible, and prognosis is generally poor [6].

Given the high risk of HCC recurrence, risk stratification is of major clinical interest. In our cohort, explant pathology was a strong predictor of HCC recurrence, with high recurrence rates observed in patients beyond the MC based on explant pathology. However, recurrence rates were still substantial among patients within the MC, underscoring the need for additional prediction tools. We, therefore, applied the recently described RETREAT score, which in our cohort was able to effectively stratify patient risk, with low recurrence rates observed in patients with RETREAT scores ≤2. Our study did identify an important caveat in applying the RETREAT score: while low RETREAT scores were able to identify a large subset at very low risk of HCC recurrence among the subgroup of patients within the MC, predictive performance was markedly reduced in patients beyond the MC based on explant pathology. These findings suggest that the RETREAT score should not be used in the latter subgroup. This subgroup is still substantial even in the age of high quality imaging [11,20], which was confirmed in our cohort. The proportion of patients beyond the MC on explant was equally distributed over the total study period. In our cohort, a RETREAT score ≤2 identified 122 of 203 patients (60.1%) who could be exempt from surveillance based on a very low (<3%) recurrence risk at 10 years post-LT. It is important to note that our RETREAT cut-off (≤2) is different from that reported by Mehta et al., who restricted the group who could be exempt from surveillance to those with a score of 0. The difference may be partly accounted for by the concomitant assessment of the MC in the explant: in the development cohort, validation cohort, and United Network for Organ Sharing (UNOS) validation, respectively, 22.1%, 37.3%, and 14.2% of the total study cohort were beyond the MC based on explant [10,11].

This study confirms the predictive performance of the RETREAT score in a different patient population. The development and (UNOS) validation study of the RETREAT score consisted of a highly pre-treated population. Over 90% of the patients had undergone LRT pre-LT [10,11], compared to 74.9% in our cohort. In our cohort, complete response to LRT was associated with a negligible risk of recurrence after transplantation. Another important difference compared to previous research on the RETREAT score is the different underlying etiology in our study population. Previous RETREAT studies enrolled patients predominantly (56–63%) infected with HCV, whereas HCV was a minority in our cohort. Since both LRT and the cause of underlying liver disease may influence tumor characteristics on explant and recurrence risk, it is encouraging to note that the predictive performance of RETREAT was maintained in our cohort. 

The overall survival in our cohort is comparable to the estimated overall survival of patients post-LT for the diagnosis of HCC in the European Liver Transplant Registry [21]. 

Some limitations of this study should be acknowledged. One is the relatively limited sample size. Secondly, our institution used a restrictive postoperative surveillance schedule. This might have delayed the HCC recurrence diagnosis. Third, we have to acknowledge the intrinsic limitation of the RETREAT score. The score is only useful post-liver transplant for the risk stratification of recurrence and is not useful for clinical decision making in a preoperative setting. 

The findings from this study may have important clinical implications. First, a subgroup of patients may be identified, 60.1% of the total cohort, that will likely experience marginal benefit from surveillance given the low recurrence risk. All other patients are at higher risk of HCC recurrence and could potentially benefit from enrolment in a surveillance program. However, the findings from this study suggest that if surveillance is undertaken, this likely involves extensive body imaging for at least five years post-transplant, with the effect of surveillance on patient survival still uncertain. 

## 5. Conclusions

In conclusion, low RETREAT scores identify patients at low risk of HCC recurrence after LT in patients within the MC based on explant pathology. These findings may be used to inform patients of recurrence risk and as a basis for studies on surveillance strategies.

## Figures and Tables

**Table 1:** Cohort characteristics.

Characteristics	*n* = 203
Median follow up time (months) (IQR)	26.8 (7.2–60.7)
Median age at LT (years), (IQR)	61.2 (54.8–65.2)
Male sex, %	162 (79.8)
Median waiting time to LT (months), (IQR)	7.7 (3.8–10.8)
Median lab MELD Score on LT, (IQR) (*n* = 188)	11 (8–15)
LRT pre LT ^a^, %	153 (75.4)
Resection pre LT, %	17 (8.4)
RFA	95 (46.8)
TACE/TARE	71 (35.0)
MWA	20 (9.9)
PEI	5 (2.5)
ILC	3 (1.5)
Stereotactic radiotherapy	2 (1.0)
Etiology of liver disease, %	
Hepatitis B	28 (13.8)
Hepatitis C	42 (20.7)
NAFLD	25 (12.3)
Alcoholic Liver Disease	33 (16.3)
Others & cryptogenic liver disease	48 (23.6)
Combined etiology	26 (12.8)

^a^ 18 patients were successfully downstaged pre LT. Downstaging is defined as a reduction in the number and size of viable tumors to within the Milan criteria. Abbreviations: IQR, interquartile range; LT, liver transplantation; LRT, locoregional therapy; MELD, Model for End Stage Liver Disease; MWA, microwave ablation; NAFLD, non-alcoholic fatty liver disease; PEI, percutaneous ethanol injection; RFA, radiofrequency ablation; TACE, transarterial chemoembolization; TARE, transarterial radioembolization.

**Table 2:** Points distribution of the RETREAT score for the overall cohort, recurrences, and no recurrences.

Predictor	RETREAT Points	Overall (*n* = 203) (%)	Recurrence (*n* = 27) (%)	No Recurrence (*n* = 176) (%)
Last AFP pre LT (µg/L)				
0–20	0	160 (78.8)	15 (55.6)	145 (82.4)
21–99	1	19 (9.4)	1 (3.7)	18 (10.2)
100–999	2	19 (9.4)	7 (25.9)	12 (6.8)
≥1000	3	5 (2.5)	4 (14.8)	1 (0.6)
Microvascular invasion	2	45 (22.2)	16 (59.3)	29 (16.5)
Largest viable tumor diameter (cm) plus number of viable tumors				
0 *	0	40 (19.7)	0 (0.0)	40 (22.7)
1.1–4.9	1	105 (51.7)	10 (37.0)	95 (54.0)
5.0–9.9	2	53 (26.1)	14 (51.9)	39 (22.2)
≥10	3	5 (2.5)	3 (11.1)	2 (1.1)

* No viable tumor on explant pathology. Abbreviations: AFP, α-fetoprotein; LT, liver transplantation.

## Data Availability

Due to the nature of this research, participants of this study did not agree for their data to be shared publicly, so supporting data is not available.

## Supplementary Materials

The following are available online at https://www.mdpi.com/article/10.3390/cancers14030630/s1, Figure S1: Study cohort flow diagram. Figure S2: Comparable recurrence risk in patients within the Milan criteria on explant and a high RETREAT score ^a^ and patients beyond the Milan criteria ^b^ on explant. Table S1: Individual patients characteristics of patients with HCC recurrence after LT.

## Abbreviations

AFP, α-fetoprotein; HBV, hepatitis B virus; HCV, hepatitis C virus; HCC, hepatocellular carcinoma; LT, liver transplantation; LRT, locoregional therapy; MELD, Model for End-Stage Liver Disease; MC, Milan criteria; MWA, microwave ablation; NAFLD, non-alcoholic fatty liver disease; PEI, percutaneous ethanol injection; RETREAT, Risk Estimation of Tumor Recurrence After Transplant; RFA, radiofrequency ablation; TACE, transarterial chemoembolization; TARE, transarterial radioembolization.
