# Supplementary material for: External Validation of the RETREAT Score for Prediction of Hepatocellular Carcinoma Recurrence after Liver Transplantation
Source: Cancers (Basel). 2022 Jan 27;14(3):630. doi: 10.3390/cancers14030630 (PMC8833722; doi:10.3390/cancers14030630)

Supplementary Figure S1. Study cohort flow diagram. Abbreviations: HCC, hepatocellular carcinoma; LRT, Locoregional therapy; LT, liver transplantation.

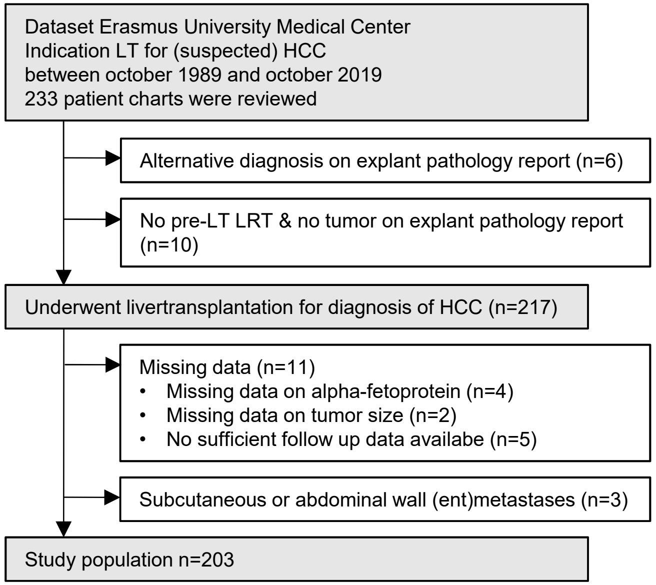

Supplementary Figure S2. Comparable recurrence risk in patients within Milan Criteria on Explant and a high RETREAT score[a] and patients beyond Milan Criteria[b] on Explant. [a] RETREAT Score: AFP at LT, microvascular invasion and sum of largest viable tumor diameter and number of tumors. [b] Milan criteria (MC): 1 tumor ≤5 cm, or 3 tumors ≤3 cm each.
Abbreviations: HCC, hepatocellular carcinoma; LT, liver transplantation.

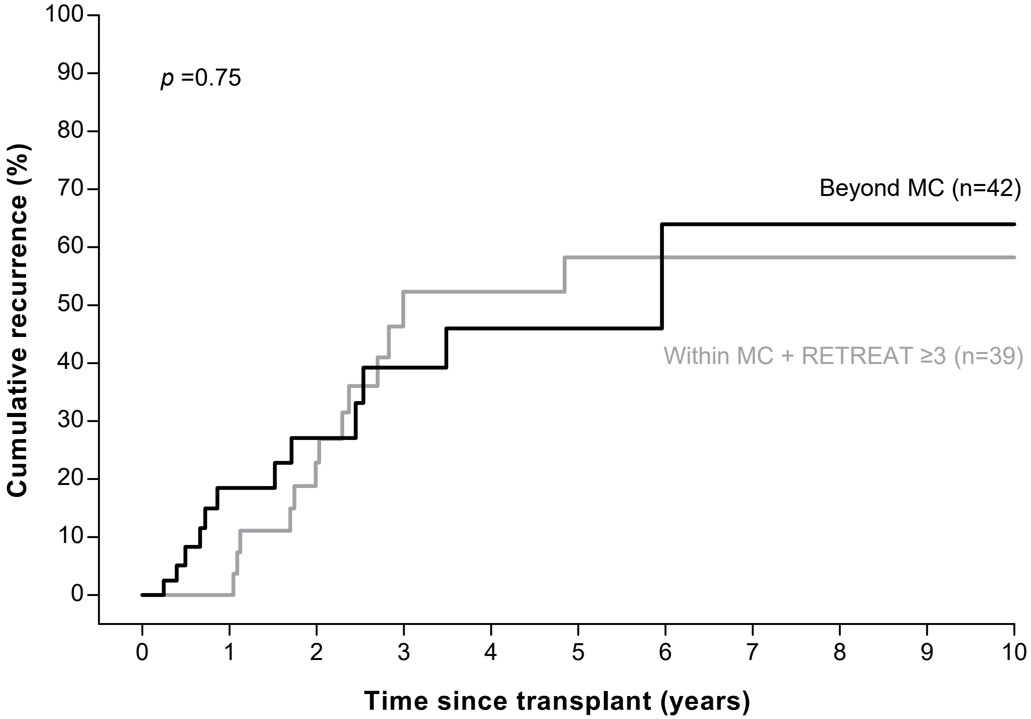

Supplement: Supplementary file 1 [file cancers-14-00630-s001.zip › Supplementary Figures.pdf]
