# Supplementary material for: External Validation of the RETREAT Score for Prediction of Hepatocellular Carcinoma Recurrence after Liver Transplantation
Source: Cancers (Basel). 2022 Jan 27;14(3):630. doi: 10.3390/cancers14030630 (PMC8833722; doi:10.3390/cancers14030630)
Supplement: Supplementary file 1 [file cancers-14-00630-s001.zip › Supplementary Table S1. Individual patients characteristics of patients with HCC recurrence after LT_revisions.pdf]

Supplementary Table S1. Individual patients characteristics of patients with HCC recurrence after LT.

	Etiology	AFP points	Micro-invasion	Sum greatest viable tumor and number of tumors	Total RETREAT score	LRT	Time between LT and recurrence[a] (months)	Recurrence	Location of recurrence	Recurrence AFP (µg/l)	Time between recurrence and death (months)	Oncologic treatment
**1**	Others & Cryptogenic liver disease	0	0	3	3	no	29.4	Clinical consensus	Liver, lung	8	1.3	No treatment
**2**	Hepatitis C	3	0	2	5	no	18.0	Clinical consensus	Liver	1000	10.4	No treatment
**3**	Hepatitis C	2	0	1	3	no	24.7	Pathology	Ovarian	9393	13.7	Ovarian extirpation
**4**	Hepatitis B	3	0	2	5	no	12.5	Clinical consensus	Liver, lung	3300	10.9	No treatment
**5**	Alcoholic Liver Disease	3	2	2	7	yes	8.7	Pathology	Liver	28480	2.7	No treatment
**6**	Hepatitis B	0	2	2	4	no	13.5	Clinical consensus	Adrenal gland, liver, lung	33	1.6	No treatment
**7**	Hepatitis B	0	0	2	2	yes	30.4	Clinical consensus	Liver, lung	430	5.1	No treatment
**8**	Others & Cryptogenic liver disease	2	0	2	4	yes	13.1	Clinical consensus	Liver	21887	8.6	RFA liver
**9**	Hepatitis B	3	2	2	7	yes	18.2	Pathology	Lymph node (mediastinal)	84775	4.9	No therapeutic options
**10**	Others & Cryptogenic liver disease	2	0	1	3	yes	30.9	Clinical consensus	Bone	987	21.0	Radiotherapy
**11**	Hepatitis B	1	2	2	5	no	10.3	Clinical consensus	Bone, Lung	7591	0.5	No treatment
**12**	NAFLD	0	0	2	2	no	71.5	Pathology	Liver, peritoneal	254	7.9	Sorafenib

**13**	Hepatitis C	2	0	2	4	yes	41.9	Pathology	Bone	Unknown	0.5	(palliative) Radiotherapy
**14**	NAFLD	2	2	2	6	yes	20.5	Clinical consensus	Bone	Unknown	10.8	Radiotherapy + Sorafenib
**15**	NAFLD	2	2	2	6	yes	20.9	Clinical consensus	Liver, lung, peritoneal	4000	13.3	Unknown
**16**	Hepatitis C	2	2	1	5	yes	58.1	Pathology	Adrenal gland, liver, lung	25	28.3	Sorafenib
**17**	Hepatitis C	0	0	1	1	yes	17.4	Clinical consensus	Adrenal gland, liver, peritoneal	106	6.6	No treatment
**18**	Others & Cryptogenic liver disease	0	2	2	4	no	4.8	Clinical consensus	Bone, lymph nodes (mesenteric), lung	9	3.6	(palliative) Radiotherapy
**19**	Others & Cryptogenic liver disease	0	0	1	1	yes	31.8	Pathology	Liver, lymph nodes (para aortic, iliac), rectal	12	7.4	Sorafenib
**20**	Hepatitis B	0	2	1	3	yes	24.3	Clinical consensus	Liver, lung, lymph nodes (mediastinal)	Unknown	11.6	No treatment
**21**	Hepatitis C	0	2	1	3	no	20.4	Pathology	Lung	8	29.1	Regorafenib
**22**	Hepatitis C	0	2	1	3	yes	33.8	Pathology	Lung	Unknown		Sorafenib
**23**	Combined etiology	0	2	1	3	no	33.9	Pathology	Adrenal gland	3		Unknown
**24**	Alcoholic Liver Disease	0	2	1	3	yes	24.5	Pathology	Lung	4		Lobectomy, stereotactic radiotherapy
**25**	Alcoholic Liver Disease	0	2	2	4	yes	8.0	Pathology	Bone	2796	7.0	Radiotherapy
**26**	Combined etiology	0	2	3	5	yes	5.9	Pathology	Liver, omental , peritoneal	43		Sorafenib
**27**	Alcoholic Liver Disease	0	2	3	5	yes	2.9	Pathology	Lung, lymph node	1988	5.1	Sorafenib

[a]Diagnosis of HCC recurrence was based on histopathology reports or clinical consensus

Abbreviations: AFP, α-fetoprotein; LRT, Locoregional therapy; LT, liver transplantation; NAFLD, Non alcoholic fatty liver disease; RETREAT, Risk Estimation of Tumor Recurrence After Transplant, RFA , Radiofrequency Ablation.
